# From Light Harvesting to Grain Filling: Chlorophyll Fluorescence, Pigment Composition, and Oxidative Status as Discrete Yield Determinants in Rye

**DOI:** 10.3390/plants14243746

**Published:** 2025-12-09

**Authors:** Maria Duszyn, Paweł Burdiak, Joanna Dąbrowska-Bronk, Anna Rusaczonek, Muhammad Kamran, Roshanak Zarrin Ghalami, Alina Majnert, Jarosław Bojarczuk, Piotr Gawroński, Stanisław Karpiński

**Affiliations:** 1 Department of Plant Genetics, Breeding, and Biotechnology, Institute of Biology, Warsaw University of Life Sciences, 02-776 Warsaw, Poland; maria_duszyn@sggw.edu.pl (M.D.); pawel_burdiak@sggw.edu.pl (P.B.); muhammad_kamran@sggw.edu.pl (M.K.); roshanak_zarrin_ghalami@sggw.edu.pl (R.Z.G.); piotr_gawronski1@sggw.edu.pl (P.G.); 2 Department of Botany and Plant Physiology, Institute of Biology, Warsaw University of Life Sciences, 02-776 Warsaw, Poland; joanna_dabrowska@sggw.edu.pl (J.D.-B.); anna_rusaczonek@sggw.edu.pl (A.R.); 3 Plant Breeding Smolice Ltd., IHAR Group, 63-740 Smolice, Poland; a.majnert@hrsmolice.pl (A.M.); bojarczuk@hrsmolice.pl (J.B.)

**Keywords:** hybrid breeding, early-stage phenotyping, non-photochemical quenching, photosynthesis and yield, reactive oxygen species, photosynthetic maximal efficiency

## Abstract

Improving rye (*Secale cereale*) yield under increasing climatic stress remains a major challenge for sustainable cereal production. We examined whether early-vegetative physiological, biochemical, and molecular traits can predict final grain yield in hybrid-breeding components. Across three consecutive seasons, 14 genotypes were evaluated under controlled cold-greenhouse conditions for chlorophyll fluorescence, pigment content, hydrogen peroxide (H_2_O_2_), salicylic acid (SA) levels, and the expression of selected antioxidant and defence-related genes, and these traits were related to yield components. Across years, photosynthetic efficiency (*F*_v_/*F*_m_, Rfd), chlorophyll content, and foliar H_2_O_2_ emerged as the most consistent predictors of kernel mass, spike number, and kernel number. In contrast, non-photochemical quenching, SA, and carotenoid contents showed weak or inconsistent relationships with yield. These findings indicate that light-harvesting capacity, PSII performance, and oxidative balance are central to reproductive success in rye. The stability of these trait–yield correlations across three seasons provides the basis for a physiological robustness index for hybrid rye, with predictive models achieving accuracies up to R = 0.51. This work demonstrates the potential of using a compact set of early-stage, high-throughput physiological traits to accelerate selection for stress-resilient, high-yielding rye cultivars.

## 1. Introduction

Cereals are staple foods worldwide, and improving their performance is crucial for food security, economic development, and environmental sustainability. Breeding efforts focus on developing high-yielding, nutritious, and resilient varieties with improved resistance to biotic and abiotic stresses such as drought, heat, and salinity, which are increasingly important under climate change [1]. Nutritional improvements, including increased protein content and biofortification with vitamins and minerals, are also important targets [2]. Together, these approaches help reduce malnutrition, particularly in developing regions where cereals are dietary staples.

Rye (*Secale cereale* L.) is an important cereal valued for its resilience and adaptability to poor soils and cold climates. It is cultivated as grain, forage, and cover crop. Its outbreeding nature and genetic diversity make rye especially suited for hybrid breeding, systematically applied since the late 20th century to increase yield and stress tolerance [3]. Strong heterosis in hybrid rye depends on genetically distinct parental pools, as confirmed by nucleotide polymorphism (SNP)-based studies [4]. High-yielding hybrid rye breeding relies on cytoplasmic male sterility (CMS) systems, with fertility restoration controlled by nuclear genes such as *Rfp3*. Advances in genomic tools allow breeders to optimise breeding and avoid linkage drag effects that reduce yield [3,4]. Current rye breeding targets include yield stability, drought tolerance, disease resistance (e.g., ergot, brown rust, mildew), and grain quality traits, while high biomass and methane yield also make rye valuable for bioenergy production [5].

Crop breeding began 10,000–12,000 years ago, when humans first selected edible plants for cultivation. Early breeding relied on choosing naturally mutated plants with beneficial traits, which over millennia led to significant divergence between modern crops and their wild ancestors [6]. The discovery of Mendelian genetics enabled advanced methods such as induced mutation, polyploidy, and hybrid breeding [7]. Since the mid-20th century, molecular tools including marker-assisted selection and pedigree analysis have increased the precision of trait selection [8]. More recently, high-throughput omics, genome editing, and machine learning have revolutionised crop improvement by enhancing trait mapping, phenotyping, and data analysis [9,10,11]. These innovations have initiated a new era of data-driven, precision breeding.

The precision and efficiency of hybrid breeding can be improved by integrating physiological, biochemical, and transcriptomic data. These methods support early prediction of parental line value and hybrid performance. Physiological traits and metabolite profiles reflect plant responses to environmental conditions and are useful predictors of yield potential. For example, nitrogen-use efficiency in sesame was dissected using physiological and molecular analyses, revealing key metabolites and proteins responsive to nitrogen fertilization levels that correlated with yield traits [12]. Metabolite profiling also helps identify compounds such as amino acids, carbohydrates, and secondary metabolites that vary with genotype and environmental factors, providing predictive markers for yield potential and stress tolerance. Transcriptomic profiling provides a dynamic view of gene regulation patterns and complements genomic markers. For instance, in maize, transcriptome-based distances correlated strongly with hybrid performance, enabling early selection of promising inbred lines without field testing [13]. Combining transcriptomic and genomic data further improves predictive accuracy for complex traits such as dry matter yield. Multi-omics tools reduce reliance on resource-intensive field trials and allow breeders to efficiently identify optimal parental combinations from large heterotic pools [14].

In cereals, telemetry and biochemical markers have also been shown to correlate with yield through physiological and environmental interactions. Telemetry includes remote sensing and high-throughput phenotyping methods that monitor growth, biomass, and physiological status in real time. Traits such as canopy temperature, biomass accumulation, and chlorophyll content are closely linked to yield potential [15]. In rye, biochemical markers related to yield have been studied primarily through genome-wide association studies (GWAS) and quantitative trait loci (QTL) mapping that link genetic markers with agronomic traits including grain yield, plant height, heading date, and grain quality. Biochemical markers including enzyme activities, metabolite concentrations, and nutrient content in grains correlate with yield components [16]. For example, grain yield positively correlates with grain filling period, number of productive tillers, spike length, number of kernels per spike, thousand kernel weight, and biomass yield [17]. Biochemical markers related to nitrogen use efficiency, photosynthesis, and photorespiration also influence yield, as nitrogen uptake and metabolism strongly affect grain development [18]. In addition, grain micronutrient concentrations (e.g., iron and zinc) show variable correlations with yield depending on environmental conditions, highlighting the complexity of breeding for both nutrition and productivity [19]. Pigment composition is also relevant: while high pigment levels are generally beneficial, relative ratios such as chlorophyll *a:b* or chlorophyll/carotenoid provide better indicators of stress resistance and productivity [20,21]. Molecular markers linked to candidate genes identified through GWAS and transcriptomic analyses further support marker-assisted selection for yield and stress tolerance [16,22]. Functional markers, which are closely associated with phenotypic traits, enhance selection efficiency by enabling precise gene targeting in breeding programmes. Integrating genomic, transcriptomic, and metabolic markers provides a comprehensive selection index that captures the multilayered biological basis of heterosis, thus improving the prediction of hybrid performance and heterosis levels [23].

Among telemetric markers, chlorophyll fluorescence is one of the most extensively studied, as it reflects photosynthetic efficiency and has been linked to yield in wheat, maize, and oats. Fluorescence parameters such as *F*_v_/*F*_m_ or non-photochemical quenching (NPQ) serve as proxies for energy conversion efficiency, linking physiological status to biomass accumulation and yield. Field studies in durum wheat showed correlations between fluorescence and yield [24], while in winter wheat, fluorescence traits correlated with gas exchange and yield [25]. At larger scales, satellite-based solar-induced fluorescence has been used to predict wheat and maize yields, offering a powerful approach under climate variability [26]. In oats, doubled haploid lines with higher fluorescence efficiency were linked to greater grain yield and biomass [27]. Similarly, remote sensing of absorbed photosynthetically active radiation (fAPAR) correlates with cereal yields across Europe, further supporting the use of chlorophyll fluorescence as a valuable telemetric marker [28].

In this study, we apply a unique multi-year dataset integrating a broad range of parameters, including chlorophyll fluorescence indicators (e.g., *F*_v_/*F*_m_, NPQ, qP), pigment composition (e.g., chlorophylls, carotenoids, violaxanthin), oxidative stress markers (e.g., hydrogen peroxide, salicylic acid), and expression levels of selected genes. These parameters were measured in rye leaves at the early developmental stage and integrated with final yield data for all parental genetic components used in hybrid breeding. We hypothesise that early-stage telemetric, biochemical, and transcriptomic markers assessed during vegetative growth are significantly correlated with yield traits such as kernel mass and spike number, and that predictive models integrating these markers can guide breeding of high-yielding rye hybrids. In this study, all measurements were conducted under cold-greenhouse conditions to provide a controlled and reproducible setting for assessing early-stage physiological, biochemical, and transcriptomic traits. This environment was chosen to minimise external variability and highlight genotype-dependent differences. Although field trials were beyond the scope of this work, the greenhouse analyses represent an initial screening step, forming a basis for future validation of identified markers under field conditions.

## 2. Results

### 2.1. Trait Variation Across Rye Genotypes

To investigate links between physiological markers and yield traits in rye (*Secale cereale*), we analysed a panel of 14 genotypes, hereafter referred to as “objects” (Table 1). These represented diverse breeding components used in the development of elite F_1_ hybrids (TURF1, StachF1, TD525F1), including three elite final hybrids (FHs), three fertility restorers (RFs), three single-cross hybrids (SCs), three complementary lines (CLs), and two male-sterile lines (MSs). All genotypes were cultivated under cold-greenhouse conditions for three consecutive years (2022–2024). Plants were sampled at two developmental stages: the early vegetative phase (end of tillering, BBCH 30–39, up to the flag leaf unrolled stage) and the end of reproductive growth. Four yield traits were measured (total kernel mass [Mass], spike number [No. spikes], kernel number [No. kernels], and thousand-kernel weight [TKW]) alongside 21 physiological parameters. The latter included chlorophyll *a* fluorescence parameters (six traits: minimal fluorescence [*F*_0_], maximal fluorescence [*F*_m_], maximum quantum yield of PSII [*F*_v_/*F*_m_], non-photochemical quenching [NPQ], photochemical quenching [qP], and vitality index (chlorophyll fluorescence decrease ratio) [Rfd]); photosynthetic pigments (10 traits: violaxanthin [Viol], antheraxanthin [Anth], lutein [Lut], zeaxanthin [Zea], chlorophyll *a* [Chl *a*], chlorophyll *b* [Chl *b*], β- carotene [β-Crt], total chlorophyll content [Chl tot.], chlorophyll *a*/*b* ratio [Chl *a*/*b*], and de-epoxidation state [VAZ]); biochemical markers (hydrogen peroxide [H_2_O_2_] and salicylic acid [SA]); and transcript levels of three genes: *Lesion* *Simulating Disease 1* (*ScLSD1*), *Ascorbate Peroxidase 1* (*ScAPX1*), and *Enhanced Disease Susceptibility 1* (*ScEDS1*) (Table 2).

For each parameter, we calculated the Relative Fold Change (RFC), defined as the ratio of the maximum to minimum value across genotypes. Substantial variation was detected (Table 2). Yield traits were most divergent: total kernel mass varied 15.4-fold (from 21.9 g in PB31 to 1.4 g in PB35) and kernel number 13.1-fold (from 596 to 45) (Figure S1). In contrast, physiological parameters were generally more stable, with RFC values of 1.2–2.1. The most invariant traits were photochemical quenching and the chlorophyll *a*/*b* ratio (RFC ≈ 1.2) (Figure S1), whereas pigments varied moderately (RFC 1.3–1.4). VAZ exhibited greater plasticity (RFC = 1.62). Among biochemical and molecular markers, *ScAPX1* expression showed pronounced variation (RFC = 3.87), suggesting large genotypic differences in antioxidant capacity, while *ScLSD1* and *ScEDS1* were more moderate (RFC 1.65–1.87), and could be related to differential regulation of cell death and defence signalling.

Nonparametric factorial ANOVA (aligned rank transform, ART) confirmed significant differences among genotypes for most traits (Table 3). Traits with high RFC values, such as kernel number and *ScAPX1* expression, also showed strong genotype effects, indicating genetically structured variation. Year effects were significant for all parameters, particularly *F*_v_/*F*_m_ and the Chl *a/b* ratio, reflecting environmental modulation. Moreover, significant genotype × year interactions were observed for most traits, except kernel number, indicating differential genotypic responses to growth conditions. Overall, yield traits were highly divergent, while physiological and molecular traits were more constrained but still captured meaningful genetic and environmental variation.

To complement these nonparametric tests, we additionally estimated variance components using linear mixed-effects models (REML). The mixed-model results (Table S1) were consistent with the ART analysis: total kernel mass and kernel number remained strongly genotype-determined (≈49–51% of variance), whereas TKW showed the largest genotype × year interaction (42%), reflecting high environmental sensitivity. For Rfd, the REML model confirmed the absence of a year effect observed in ART, with the year component effectively contributing 0% of variance. Together, these analyses support the conclusion that yield traits are predominantly shaped by genetic differences, with variable but interpretable environmental modulation across traits.

### 2.2. Correlations Among Physiological, Molecular, and Yield Traits

**Figure 1:**
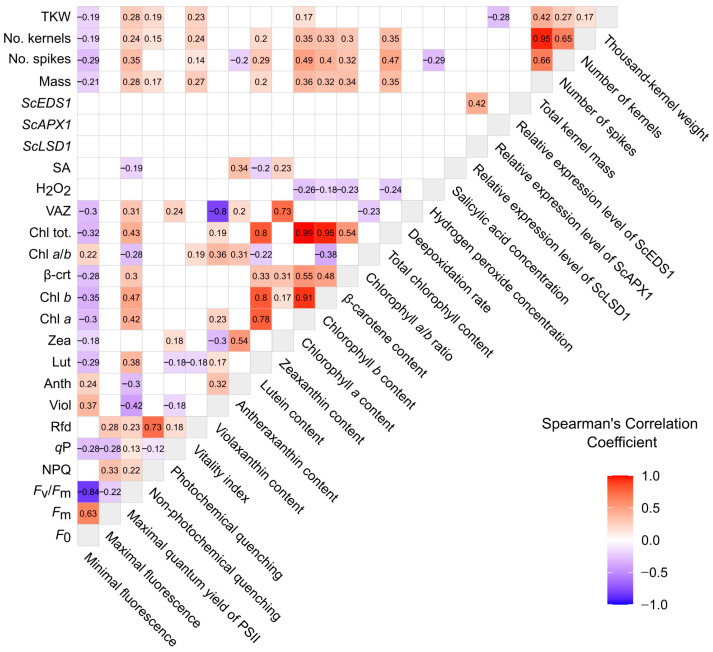
The heatmap showing Spearman’s correlation coefficients of physiological, molecular, and yield traits. Only statistically significant correlations (*p* < 0.01) are shown; nonsignificant values are left blank. The colour intensity indicates the strength and direction of the correlation, with positive and negative associations shown in red and blue, respectively. For detailed information on number of observations (N) and FDR values please see the Supplementary Materials File S2.

Spearman correlation coefficients were calculated to assess trait relationships (Figure 1). As expected, most significant correlations occurred within parameter groups. Among fluorescence traits, Rfd and NPQ were positively correlated (R = 0.73, FDR < 2.2 × 10^−16^), whereas *F*_v_/*F*_m_ and *F*_0_ showed a strong negative correlation (R = −0.84, FDR < 2.2 × 10^−16^). Pigment traits also correlated strongly, with Chl *a* and Chl *b* tightly linked (R = 0.91, FDR < 2.2 × 10^−16^), while VAZ correlated negatively with violaxanthin content (R = −0.79, FDR < 2.2 × 10^−16^). Yield traits followed a similar pattern, with kernel number and total kernel mass almost perfectly correlated (R = 0.95, FDR < 2.2 × 10^−16^). Among molecular markers, *ScEDS1* and *ScLSD1* expression correlated positively (R = 0.42, *p* = 1.7 × 10^−4^), consistent with their known roles in cell death regulation in biotic and abiotic stress responses [29,30] (Figure S2).

**Figure 2:**
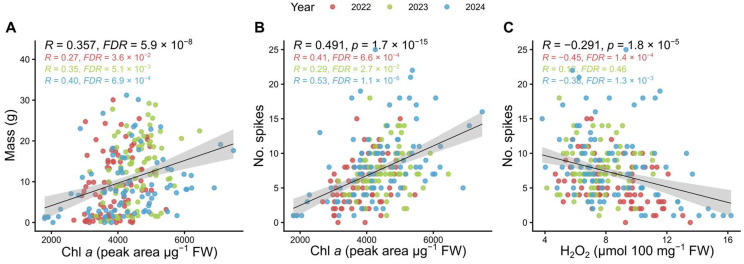
Correlations between selected biochemical markers and yield-related traits across years. Scatterplots show relationships between (**A**) chlorophyll *a* content and total kernel mass, (**B**) chlorophyll *a* content and spike number, and (**C**) hydrogen peroxide content and spike number. Points represent individual observations, colour-coded by year. Black lines indicate linear regression fits with 95% confidence intervals. Spearman’s rank correlation coefficients (*R*) and FDR-adjusted significance values (Benjamini–Hochberg) (*FDR*) are shown within each panel (black: combined dataset; coloured: individual years).

Cross-group correlations were generally weaker but several notable links were observed. Chlorophyll *a* correlated with total kernel mass (R = 0.36, FDR = 5.9 × 10^−8^) and spike number (R = 0.49, FDR = 1.7 × 10^−15^) (Figure 1 and Figure 2), suggesting that greater allocation to light-harvesting complexes supports reproductive development. *F*_v_/*F*_m_ correlated positively with yield traits—total kernel mass (R = 0.28, FDR = 3.3 × 10^−9^), spike number (R = 0.35, FDR = 1.5 × 10^−14^), and kernel number (R = 0.24, FDR = 8.0 × 10^−7^)—indicating that higher PSII efficiency enhances performance (Figure S2). β-carotene also correlated with kernel mass (R = 0.34, FDR = 3.2 × 10^−7^), consistent with carotenoid-mediated photoprotection supporting productivity under variable light (Figure S2).

These correlations remained significant when data from individual years were analysed separately, although significance was stronger in the combined dataset, reflecting greater statistical power. This consistency across years indicates that the observed associations reflect intrinsic physiological relationships rather than experiment-specific artefacts. One additional case was the negative correlation between H_2_O_2_ content and spike number (R = −0.29, FDR = 1.8 × 10^−5^), which was significant only in 2022 and 2024 (Figure 2), although our greenhouse climate data showed there were no clear temperature or irradiance differences among years.

Together, these results demonstrate numerous significant correlations both within and across parameter groups. Importantly, several physiological (Chl *a*, *F*_v_/*F*_m_, β-carotene) and biochemical (H_2_O_2_) markers were consistently linked to yield, identifying them as promising early-stage indicators for use in breeding programmes and predictive models of productivity.

### 2.3. Multivariate Modelling of Yield Prediction

**Figure 3:**
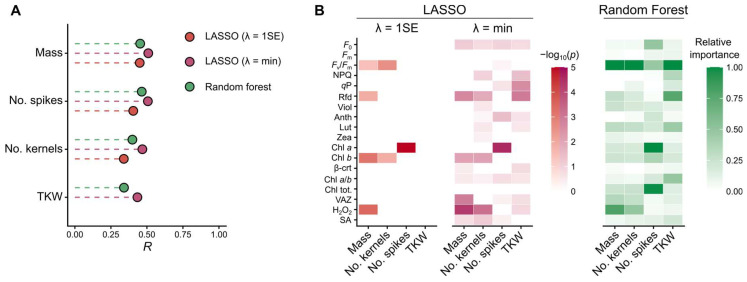
Comparative performance and feature relevance of LASSO and Random Forest across yield traits. (**A**) Comparative performance across yield traits, regularization settings, and model types. Points show R from models refitted with predictors selected by LASSO or Random Forest. LASSO is shown for two penalty choices: λ = 1SE (one-standard-error rule; sparser models) and λ = min (minimum cross-validated error). Higher R indicates better fit. (**B**) Heatmaps summarizing the relevance of selected markers for each yield trait. For LASSO, fill encodes –log_10_(*p*) from the refitted linear models (faceted by λ = 1SE and λ = min); for Random Forest, fill encodes normalised variable importance. Rows are physiological/biochemical markers; columns are yield traits; higher values indicate stronger association/contribution.

Given that several early-stage markers correlated with yield traits, we next tested whether multivariate models could improve predictive accuracy. To this end, we fitted penalised linear models using the least absolute shrinkage and selection operator (LASSO) and, in parallel, nonparametric Random Forest (RF) regressions. Variables with high proportions of missing data (including gene expression markers) were excluded. The final dataset comprised 18 physiological predictors and four yield traits, with 201 complete observations retained for analysis (Figure 3). To verify that the use of complete cases did not bias model performance, we additionally repeated all multivariate analyses on a missForest-imputed full dataset (N = 570). As described below, these sensitivity analyses yielded results highly consistent with the complete-case analyses.

LASSO models achieved prediction accuracies of up to R = 0.51, depending on the penalty parameter λ. The more inclusive λ = min models provided higher predictive power, whereas the stringent λ = 1SE criterion produced simpler but more stable models. Under λ = 1SE, total kernel mass was predicted by *F*_v_/*F*_m_, Rfd, Chl *b*, and H_2_O_2_; spike number by Chl *a*; and kernel number by *F*_v_/*F*_m_ and Chl *b*, while no predictors were retained for TKW. In contrast, λ = min models admitted additional predictors, including VAZ, NPQ, qP, and Anth. These selections highlight the importance of light-harvesting efficiency (*F*_v_/*F*_m_, Rfd, Chl pigments) and redox balance (H_2_O_2_) for assimilation availability and reproductive output, while also implicating photoprotective adjustments (xanthophyll-cycle activity, NPQ) in yield variation. Overall, chlorophyll content, photosynthetic efficiency, and oxidative-stress markers emerged as the most consistent predictors across genotypes. Importantly, repeating the LASSO analyses on the imputed dataset produced nearly identical predictor sets and similar R values, with trait ranking unchanged. This confirms that the linear predictive relationships were robust to missing-data handling.

RF models achieved comparable prediction accuracies: R = 0.45 for total kernel mass, R = 0.46 for spike number, R = 0.40 for kernel number, and R = 0.34 for TKW. Variable-importance analysis again highlighted *F*_v_/*F*_m_, particularly for kernel number and TKW, underscoring the role of PSII efficiency in sink establishment and grain filling. H_2_O_2_ was a strong contributor to predictions of total kernel mass and kernel number, consistent with oxidative homeostasis acting as a constraint on reproductive success. Pigment traits, notably Chl *a* and Chl tot., were most influential for spike number, suggesting that genotypes investing more strongly in light capture sustain reproductive development more effectively. Photoprotective markers (NPQ, Chl *a*/*b* ratio, lutein) contributed to grain-weight prediction, reflecting the importance of balancing light capture with energy dissipation. Re-running the RF models on the imputed dataset resulted in consistently higher R values—as expected due to the larger sample size—but preserved the same trait ranking and the same dominant predictors (*F*_v_/*F*_m_, H_2_O_2_, and pigment markers). No contradictions or reversals in variable importance were observed, further supporting the robustness of the RF findings.

In addition to supervised modelling approaches (LASSO and RF), we performed a Principal Component Analysis (PCA) (Figure S3) to visualise the major axes of covariation among the physiological and biochemical markers. The first two components explained 42.3% of the variance (PC1 = 25.2%; PC2 = 17.2%). PC1 was driven primarily by chlorophylls, carotenoids, and *F*_v_/*F*_m_, representing a light-harvesting efficiency axis, whereas PC2 was dominated by xanthophyll-cycle pigments, reflecting photoprotective investment. When plant level observations were colour-coded by yield class (total kernel mass), high-yield individuals clustered toward regions of higher chlorophyll content and PSII efficiency, while low-yielding individuals aligned with increased *F*_0_. These patterns reinforce the results of the LASSO and RF analyses, which likewise identified pigment composition, *F*_v_/*F*_m_, and NPQ-related traits as key contributors to yield variation.

Together, these results indicate that both LASSO and RF consistently prioritised photosynthetic performance and stress-related markers as key drivers of yield variation. Differences in rye productivity thus appear to be shaped primarily by the efficiency of light capture and use, coupled with the ability to maintain redox balance under stress. The agreement between complete-case and imputed-data analyses across LASSO and RF models demonstrates that the inferred physiological drivers of yield are not sensitive to missing-data treatment and therefore represent stable, biologically meaningful relationships.

## 3. Discussion

### 3.1. Photosynthetic Markers That Track Yield Performance

Across years, several photosynthetic and biochemical indicators showed strong associations with yield components in rye, highlighting the central role of light capture, photochemistry, and oxidative balance in reproductive success. Leaf chlorophyll content was consistently and positively correlated with grain yield and its components (Figure 1, Figure 2 and Figure 3). Chlorophyll *a* drives reaction-centre photochemistry, while chlorophyll *b* broadens the absorption spectrum and enlarges the antenna size. Higher total chlorophyll content therefore indicates more effective light harvesting and delayed senescence, ultimately sustaining grain filling [31,32]. In cereals, simple proxies of chlorophyll status, such as SPAD, frequently correlate with yield under water-limited conditions, consistent with the idea that “stay-green” leaves support assimilation supply during grain filling [33]. Our rye dataset supports these observations, with higher chlorophyll content co-occurring with greater yield traits (Figure 1). A similar pattern is documented in other cereals, reinforcing the broader relevance of these physiological indicators. In winter wheat, drought and shading substantially reduce yield components and simultaneously depress chlorophyll fluorescence (e.g., *F*_v_/*F*_m_), highlighting the reliance of yield on sustained photochemistry [25]. Stress-exposed wheat also typically enhances antioxidant enzyme activity to counteract ROS and maintain photosynthetic function [34]. Taken together, evidence from wheat and barley mirrors the relationships observed in rye, supporting the general applicability of chlorophyll status, photochemical efficiency, and ROS management as indicators of yield potential across cereals.

In contrast, markers of stress or chronic photoinhibition were negatively associated with yield. The maximal quantum yield of PSII (*F*_v_/*F*_m_) remained near the canonical optimum (~0.8) in the highest-yielding genotypes but declined in less productive entries (Figure S4), suggesting photodamage and impaired PSII repair [35,36]. In wheat, *F*_v_/*F*_m_ measured around anthesis after severe drought predicts the magnitude of yield loss, underscoring its utility as a rapid integrative stress readout [37]. Similarly, elevated H_2_O_2_—reflecting an unfavourable ROS balance—was associated with a reduced number of spikes (Figure 2), consistent with evidence that excessive ROS impairs reproductive development and grain set under abiotic stress [38,39]. Taken together, these results support a simple physiological model: genotypes that efficiently harvest light while avoiding photo-oxidative damage are better able to sustain assimilation supply to reproductive sinks, thereby achieving higher yields.

When interpreting the correlations observed between physiological traits and yield, it is important to emphasise that these relationships do not imply direct causation. The associations identified here indicate that certain physiological, biochemical, or structural traits co-vary with yield across genotypes under controlled greenhouse conditions, but they do not demonstrate that these traits mechanistically determine yield outcomes. Many correlated traits may instead reflect shared upstream processes, coordinated developmental programmes, or genotype-specific strategies rather than causal drivers. Therefore, while the correlations highlight promising physiological markers for early screening, establishing causal pathways will require targeted functional studies and evaluation under diverse field environments.

Importantly, trait–yield relationships were stable across seasons (Figure 2 and Figure S2), suggesting they reflect genotype-intrinsic robustness rather than transient environmental effects. This pattern mirrors the “functional stay-green” paradigm in cereals, where delayed senescence and sustained photosynthetic activity contribute to yield stability across environments [31,40,41]. The repeated association of higher *F*_v_/*F*_m_ with superior yield (Figure S4) suggests that inherently stronger photoprotection and/or faster PSII repair enables resilient genotypes to avoid chronic photoinhibition during stress episodes [36]. Similarly, consistently lower H_2_O_2_ in top performers (Figure 2) indicates a durable antioxidant capacity that buffers climatic variability from year to year [38,39]. This cross-year coherence strengthens the case for using these traits as selection criteria for broad adaptability. To apply these robust trait–yield associations in breeding, reliable high-throughput phenotyping methods are essential. Advances in high-throughput phenotyping, particularly relative chlorophyll content (SPAD) measurements and hyperspectral indices, have increasingly shown that rapid, non-invasive assessments can effectively replace time-consuming pigment analyses and support large-scale selection. Hyperspectral approaches have been demonstrated to predict grain yield and nitrogen status under field conditions [42], while SPAD and hyperspectral indices provide reliable estimates of pigment content in wheat and barley, even under stress [43]. These tools form an integral part of emerging photosynthetic phenotyping pipelines aimed at identifying stress-resilient genotypes [44] and help reinforce the documented links between photosynthetic performance and yield potential in cereals [45]. Collectively, such high-throughput proxies are accelerating breeder decision-making in large-scale screening efforts [46].

### 3.2. Oxidative Balance, Reproductive Success, and Environmental Dependence

Hydrogen peroxide has a dual role in plant biology: it acts as an indispensable signalling molecule at low levels but causes cellular damage at high concentrations [38,39]. In our trials, lower H_2_O_2_ content was associated with higher spike number (Figure 2), but mostly in 2022 and 2024. After closer inspection of the greenhouse climate data, there were no clear temperature or irradiance differences among years that could explain why this relationship appeared stronger in some seasons. As a result, the observed variation in correlation strength is more likely attributable to year-specific biological noise, genotype-by-year interactions not captured by the measured environmental variables, or stochastic variation in oxidative responses, rather than distinct environmental stress patterns. The consistent direction of the relationship—higher oxidative load associated with lower reproductive output—across all three years nevertheless suggests that oxidative balance is an intrinsic component of physiological performance in rye. This aligns with evidence that excessive ROS accumulation can impair floral development, grain set, and spike emergence across cereal species [37,38]. One possible explanation is that genotypes differ in their capacity to maintain redox homeostasis even under nominally similar environmental conditions, resulting in genotype-specific thresholds of oxidative stress. Cytosolic ascorbate peroxidase 1 (APX1) has been identified as an essential H_2_O_2_ scavenger in Arabidopsis and is required to maintain oxidative balance under combined stresses [41]. However, *ScAPX1* transcript abundance did not correlate with yield in our study, suggesting that antioxidant capacity in rye may be regulated post-transcriptionally or via other enzymatic pathways. Thus, while H_2_O_2_ content itself is a useful indicator, transcript-level variation alone does not fully explain redox-related yield differences.

### 3.3. Utility for Early Phenotyping and Predictive Breeding

A key outcome of this study is the identification of a compact set of early-stage, high-throughput traits with predictive value for yield: (i) fluorescence parameters (*F*_v_/*F*_m_, Rfd); (ii) photosynthetic pigment content; and (iii) oxidative stress indicators, such as H_2_O_2_ content or the expression of antioxidant/stress-regulatory genes. Importantly, because environmental conditions did not differ substantially among the three greenhouse seasons, the predictive role of these traits appears to reflect genotype-intrinsic physiological differences rather than environment-specific responses. Future work should evaluate whether rapid, non-destructive proxies such as SPAD readings or canopy reflectance indices of chlorophyll and carotenoids can substitute for more labour-intensive HPLC-based pigment analyses, thereby enabling broader application in breeding. Evidence from wheat demonstrates that *F*_v_/*F*_m_ measured near anthesis under drought conditions predicts yield penalties [37], while SPAD and hyperspectral chlorophyll estimates reliably track physiological traits linked to yield under stress [33,47]. Incorporating such “secondary traits” into genomic prediction models could improve selection accuracy, especially in stress-prone environments, by integrating genotype × environment interactions through physiologically meaningful markers [31,32]. With the growing availability of portable fluorometers, UAV-based spectral imaging, and targeted transcript assays, the implementation of such trait panels at breeding scale is becoming increasingly feasible.

### 3.4. Limitations and Outlook

Several limitations should be acknowledged. First, correlation does not imply causation. Although physiological interpretations are compelling, mechanistic validation—for example by perturbing antioxidant capacity or antenna size in rye—would strengthen the conclusions. Available wheat/rye near-isogenic lines, such as 1RS chromosome segment variants, have demonstrated effects on ROS gradients and root development, and, together with barley chlorina mutants with reduced PSII antenna size, provide useful models to dissect ROS homeostasis, photosystem II repair mechanisms, and photoprotection in cereals [48,49,50]. Incorporating such genetic tools in future experiments would deepen understanding of photoprotection and oxidative balance linked to yield stability. Second, although we originally hypothesised that variation in correlation strength across years might reflect seasonal differences in stress exposure, our environmental assessment indicates that temperature and irradiance profiles were broadly comparable across years. Thus, year-to-year variation in trait–yield correlations likely reflects genotype-specific physiological variability, unmeasured microenvironmental differences, or sampling noise, rather than distinct seasonal stress conditions. Third, it remains unclear whether the identified predictors are applicable across the broader genetic diversity of rye. Validation in a wider panel of accessions will therefore be essential. Finally, our experiments were conducted under greenhouse conditions, and thus the trait–yield relationships identified here should be confirmed in field environments.

Despite these caveats, the consistent identification of chlorophyll content, *F*_v_/*F*_m_ (and Rfd), and H_2_O_2_ as predictors across seasons and genotypes, in contrast to other tested parameters, such as NPQ, SA, and carotenoid contents that showed weak/non-significant or inconsistent correlations with yield, supports their use in defining a “physiological robustness index” for hybrid rye improvement. Emerging remote and high-throughput phenotyping platforms, including UAV-based imaging, hyperspectral sensing, and automated pigment estimation, now offer realistic opportunities to scale these physiological indicators to field-level breeding workflows [25]. Integrating such technologies into future multi-environment trials will be essential for assessing the transferability of our findings to on-farm conditions and for enabling routine, large-scale selection based on these traits. Integrating such technologies into multi-environment trials will be essential for testing the transferability of these trait–yield relationships to field conditions. Overall, our results suggest that a small set of early physiological markers, such as chlorophyll level, PSII efficiency, and oxidative balance, could guide selection for stress-resilient, high-yielding rye. Because these relationships were observed under consistent environmental conditions, they likely reflect stable, genotype-dependent physiological differences. Field validation across diverse environments and genetic backgrounds will be essential to determine their robustness and practical value for routine selection in cereal breeding.

## 4. Materials and Methods

### 4.1. The Plant Material, Growth, and Yield Analysis

Rye (*Secale cereale*) plants representing various genotypes (Table 1) were classified according to their roles in the breeding process: final hybrids (FHs), fertility restorers (RFs), single-cross hybrids (SCs), complementary lines (CLs), and male-sterile lines (MSs) (Figure S5). Kernels were sown in October, and plants were cultivated under cold-greenhouse conditions. Harvesting was performed in July of the following year. Mean monthly temperature and irradiance values recorded in the cold greenhouse during the experiment were very similar across years. The average temperature values were as follows: January: 4.71 °C; February: 6.84 °C; March: 10.7 °C; April: 13.62 °C; May: 18.91 °C, June: 23.74 °C; July: 24.94 °C; August: 24.62 °C; September: 26.66 °C; October: 15.6 °C; November: 8.2 °C; December: 4.8 °C. The average irradiance values (in µmol m^−2^ s^−1^) were as follows: January: 45.3; February: 132.8; March: 243.1; April: 374.8; May: 469.1, June: 314.0; July: 438.3; August: 313.0; September: 459.7; October: 141.2; November: 64.6; December: 34.6. Relative humidity was not continuously monitored in this greenhouse.

Each genotype was represented by 14 randomly selected individual plants. Plants were randomised within greenhouse benches according to a complete randomised design to minimise positional effects. The same genotypes were sown in each experimental year using seed derived from the same parental seed lots, ensuring genetic consistency across years. At full maturity, all spikes were collected from each plant. The number of spikes per plant was recorded, and the kernel yield per plant was determined.

### 4.2. Chlorophyll a Fluorescence Measurement

Chlorophyll *a* fluorescence was recorded using a FluorCam 800 MF (PSI, Drasov, Czech Republic). Plants were dark-adapted for 35 min to determine F_0_ and F_m_. NPQ and qP were assessed using the instrument’s standard quenching analysis protocol as previously described [51], with leaves exposed to actinic light of 120 µmol m^−2^ s^−1^. Leaf temperature during measurements was controlled at 21 °C. A minimum of six plants per genotype was analysed.

### 4.3. Photosynthetic Pigment Analysis Using HPLC

First, 60−70 mg of frozen tissue was homogenised in a Mixer Mill MM 400 (Retsch, Düsseldorf, Germany) (5 min, 4 °C, 30 Hz), with 1 mL of cold acetone (−20 °C). The homogenate was evaporated using a Savant DNA120 SpeedVac (Thermo Scientific, Waltham, MA, USA), then dissolved in cold solvent A (acetonitrile: methanol; 90:10; *v*/*v*), and re-homogenised for 1 min. The extract was filtered through a 0.2 μm nylon filter (Whatman, Marlborough, MA, USA) into an auto-sampler vial, capped, and stored in the dark at −80 °C for HPLC analysis (Shimadzu, Kyoto, Japan). The pigments were separated on a SynergiTM 4 μm MAX-RP 80 Å LC Column 250 × 4.6 mm (Phenomenex, Torrance, CA, USA) at 30 °C. Solvent A was used for 10 min to elute all xanthophylls, followed by solvent B (methanol: ethyl acetate; 68:32; *v*/*v*) for 10 min at a flow rate of 1 mL min^−1^ to elute the rest of the pigments (chlorophylls and carotenes). The results are presented at the peak area per μg of fresh weight, following the protocol previously employed [52,53,54].

### 4.4. Analysis of H_2_O_2_ and SA Content

The concentration of hydrogen peroxide (H_2_O_2_) was assessed following the method outlined by Velikova [55], with slight adjustments, following the protocol previously employed [52,53]. First, 50–100 mg of frozen tissue was homogenised in a Mixer Mill MM 400 (Retsch, Düsseldorf, Germany) (5 min, 4 °C, 30 Hz), with 300 µL of cold 0.1% trichloroacetic acid, then centrifuged at 13,000 rpm for 15 min. The resulting supernatant was combined with 10 mM potassium phosphate buffer (pH 7.0) and 1 M potassium iodide in a 1:1:2 (*v*:*v*:*v*) ratio. The absorbance was measured at 390 nm using a microplate reader, Multiscan GO (Thermo Scientific, Waltham, MA, USA), and the H_2_O_2_ concentration was determined using an appropriate standard curve. Results were quantified and expressed as micromoles of H_2_O_2_ per 100 mg of fresh weight. Salicylic acid (SA) determination was performed according to the protocol developed by Meuwly and Métraux [56], using 2-methoxybenzoic acid and 3-hydroxybenzoic acid as internal standards. SA was eluted on a Luna^®^ 5 μm C18(2) 100 Å LC Column 150 × 4.6 mm (Phenomenex, Torrance, CA, USA) at 30 °C for 15 min using a Shimadzu HPLC System (Shimadzu, Kyoto, Japan). A low-pressure gradient system was used with 20 mM phosphate buffer (pH 2.5; adjusted with 8N HCl) and acetonitrile (75:25; *v*/*v*) at a flow rate of 1 mL min^−1^. The results were quantified and expressed as micrograms of SA per gram of fresh weight.

### 4.5. Gene Expression Analysis

RNA extraction was performed using PureLink RNA Mini Kit (Thermo Fisher Scientific, Waltham, MA, USA) according to the manufacturer’s instructions. cDNA was synthesised using a High Capacity cDNA Reverse Transcription Kit (Thermo Fisher Scientific). All PCR amplifications were run on a 7500 Fast Real-Time PCR System (Applied Biosystems) using a Power SYBR Green PCR master mix (Thermo Fisher Scientific). *ScACT* (actin) and *ScADP-RFa* (ADP-ribosylation factor) were used as reference genes to calculate relative expression [57,58]. These genes were selected as reference genes because they are commonly used and validated in *Secale cereale* and showed consistent Ct values across our sample set. All primers used in this study, together with amplicon lengths and PCR efficiencies, are listed in Table S2.

### 4.6. Data Structure and Preprocessing

All analyses were performed in R (v4.5.0). Measurements were stored in a matrix with columns for physiological, biochemical, and molecular markers and for yield traits; rows represented individual plants. Each record was annotated with year (three levels: 2022–2024), object name (genotype), and object role (FH—final hybrid; RF—restorer of fertility; SC—single-cross hybrid; CL—complementary line; MS—male-sterile line; used as metadata only). 

Outliers were removed using an interquartile range (IQR) rule applied within each parameter × year combination. For each parameter and year, we computed Q1 and Q3, set IQR = Q3 − Q1, and excluded observations outside [Q1 − 2 × IQR, Q3 + 2 × IQR]. After outlier removal the working dataset coPCR efficiencies, 570 plant-level rows (across parameters), and melt-curve specificities for all primer pairs are presented in Figure S6. For multivariate modelling (below), we restricted to complete cases across the selected predictors (18) and yield traits (4), yielding N = 201 observations common to all analyses.

To assess the potential impact of missing-data handling on multivariate inference, we additionally performed a nonparametric multiple imputation using the missForest algorithm (v1.6.1). Imputation was applied to the full post-filtering dataset (N = 570). The resulting imputed matrix, containing no missing values, was subjected to the same LASSO, and Random Forest workflows described below. These analyses served as sensitivity checks and confirmed that the multivariate structure, predictor sets, and trait ranking were highly consistent between complete-case and imputed datasets.

Spearman correlations were computed for all marker–yield pairs using the full dataset and separately within each year (2022–2024). *p*-values for all correlations (across all pairs and year strata) were adjusted using the Benjamini–Hochberg false discovery rate (FDR) procedure. Correlation plots are annotated with the corresponding FDR-adjusted *p*-values.

### 4.7. Nonparametric Factorial Tests (ART), LASSO, and Random Forest Analyses

To visualise the major axes of covariation among physiological and biochemical markers, we performed a Principal Component Analysis (PCA) using the FactoMineR package. Prior to analysis, all quantitative variables were centred and scaled to unit variance. PCA was conducted on the complete-case dataset used for multivariate modelling (18 physiological predictors; N = 201 plants). Variable loadings, contributions to principal components, and individual scores were extracted using the FactoMineR and factoextra packages. To facilitate biological interpretation, individual plants were colour-coded by yield class (low vs. high), defined using 20th and 80th percentiles of total kernel mass. PCA biplots showing variable loadings and observation scores were generated using the ggplot2.

To test effects of year, object, and year × object on each parameter, we used the Aligned Rank Transform (ART) to conduct nonparametric factorial ANOVAs. For a given parameter, rows with missing values were dropped and objects lacking observations in any year were excluded to avoid empty year × object cells. We fit response ~ year × object with art function [59]. We tabulated F- and *p*-values for year, object, and y3ar × object interaction (Table 3).

For each trait, we fit LASSO regressions (Gaussian, α = 1) with 10-fold cross-validation via cv.glmnet [60] function from glmnet package. Two regularization settings were reported: λ = min (minimum mean CV error) and λ = 1SE (largest λ within one standard error of the minimum). Predictors with non-zero coefficients at a given λ were deemed selected. For interpretability, we refit ordinary least-squares (OLS) models (with lm function) [60] using only the LASSO-selected predictors and extracted coefficients and *p*-values with broom::tidy function. Model performance for LASSO was computed in-sample from these OLS refits. For visualization, *p*-values were mapped to −log_10_(*p*); non-selected predictors were assigned 0.

To capture non-linear relations between markers and yield traits, we trained Random Forest (RF) regressors with ranger package [61]. For each trait, we performed an out-of-bag (OOB) tuning sweep over mtry ∈ {⌊√*p*⌋, ⌊*p*/3⌋, *p*}, min.node.size ∈ {3, 5, 10}, and sample.fraction ∈ {0.632, 0.8}, using num.trees = 1000, importance = “permutation”, and seed = 123. The best model minimised OOB RMSE. From the selected model, we reported OOB R^2^ and R = √R^2^ as performance metrics. Predictor relevance was quantified by permutation importance; for heatmaps, importance values were normalised within trait by the trait-specific maximum. To harmonise evaluation across methods, Random Forest predictors were converted to linear models by selecting a subset of markers by RF permutation importance and refitting OLS on that subset.

## Figures and Tables

**Table 1:** Sample information on the 14 diverse rye lines used in this study including final hybrids (FHs), restorers of fertility (RFs), single-cross hybrids (SCs), complementary lines (CLs), and male-sterile lines (MSs).

Unique Name	Role	Object Name	Component
PB21	FH	TURF1	-
PB22	RF	SR13	TURF1
PB23	SC	463P × 399N	TURF1
PB24	CL	399N	TURF1
PB25	MS	463P	TURF1 and StachF1
PB26	FH	StachF1	-
PB27	RF	5R	StachF1
PB28	SC	463P × 2130N	StachF1
PB29	CL	2130N	StachF1
PB31	FH	TD525F1	-
PB32	RF	41R	TD525F1
PB33	SC	6460P × 8639N	TD525F1
PB34	CL	8639N	TD525F1
PB35	MS	6460P	TD525F1

**Table 2:** Mean values of physiological, biochemical, molecular, and yield parameters across all analysed objects. Relative Fold Change (RFC) was defined as the ratio between the maximum and minimum values observed for a given parameter across all analysed objects. Additional information, including the number of observations (N) and standard error of the mean (SEM), is provided in Supplementary Materials File S1.

Trait	PB21	PB22	PB23	PB24	PB25	PB26	PB27	PB28	PB29	PB31	PB32	PB33	PB34	PB35	RFC
*F*_0_	100	103	126	175	113	104	118	92	101	97	98	103	113	127	1.91
*F*_m_	497	486	535	578	434	522	513	497	474	487	439	460	487	467	1.33
*F*_v_/*F*_m_	0.80	0.79	0.78	0.62	0.75	0.80	0.77	0.82	0.79	0.81	0.78	0.78	0.77	0.73	1.31
NPQ	0.98	0.81	1.07	0.86	0.68	1.14	1.00	1.05	0.77	1.03	0.72	1.08	0.87	0.90	1.69
qP	0.68	0.70	0.67	0.63	0.66	0.65	0.66	0.70	0.75	0.66	0.70	0.68	0.70	0.66	1.19
Rfd	1.61	1.49	1.70	0.97	1.21	1.83	1.47	2.03	1.43	1.54	1.31	1.55	1.55	1.12	2.09
Violaxanthin content (peak area µg^−1^ FW)	724	820	818	852	672	704	817	612	572	614	470	718	739	728	1.81
Antheraxanthin content (peak area µg^−1^ FW)	89	94	102	106	112	99	110	108	111	90	85	99	115	102	1.36
Lutein content (peak area µg^−1^ FW)	1811	1979	1926	2100	1625	1994	2116	1748	1731	1796	1806	1939	1763	1695	1.30
Zeaxanthin content (peak area µg^−1^ FW)	67	65	66	79	84	70	69	86	87	71	73	84	81	61	1.42
Chlorophyll *b* content (peak area µg^−1^ FW)	1388	1466	1490	1531	1164	1617	1613	1287	1181	1404	1392	1478	1353	1290	1.39
Chlorophyll *a* content (peak area µg^−1^ FW)	4103	4334	4618	4119	3676	5061	5004	4122	3802	4147	4164	4529	4076	3859	1.38
β-carotene content (peak area µg^−1^ FW)	1066	1251	1139	939	976	1126	1111	1007	1152	1119	1093	1215	1170	1004	1.33
Chlorophyll *a*/*b* ratio	2.96	3.00	3.11	2.73	3.14	3.16	3.08	3.23	3.25	3.00	3.00	3.07	3.02	3.01	1.19
Total chlorophyll content (peak area µg^−1^ FW)	5490	5800	6108	5650	4840	6678	6617	5410	4983	5551	5556	6007	5429	5149	1.38
De-epoxidation state	0.13	0.12	0.12	0.13	0.17	0.15	0.14	0.18	0.20	0.15	0.20	0.17	0.16	0.13	1.62
H_2_O_2_ content (µmol 100 mg^−1^ FW)	8.3	8.6	8.8	11.2	7.1	6.8	8.0	6.8	8.2	9.1	9.4	7.5	7.0	7.9	1.65
SA content (µg g^−1^ FW)	0.75	1.00	0.89	0.80	0.88	1.01	0.84	1.06	0.98	1.00	0.98	1.01	0.86	0.84	1.40
Relative expression of *ScLSD1*	1.01	0.87	0.80	0.91	0.75	0.74	0.61	0.70	0.81	0.63	0.66	0.79	0.69	0.85	1.65
Relative expression of *ScAPX1*	1.02	1.13	0.68	0.70	0.73	1.38	1.67	1.68	1.92	1.66	1.61	1.63	1.43	2.63	3.87
Relative expression of *ScEDS1*	1.01	0.69	0.92	1.03	0.77	0.81	0.81	1.12	1.22	0.78	0.77	0.86	0.65	0.98	1.87
Total kernel mass (g)	18.3	16.9	13.6	1.8	1.8	18.0	13.4	14.3	4.2	21.9	13.8	10.8	8.6	1.4	15.35
Number of spikes	7.9	8.6	10.3	2.7	7.3	8.9	8.0	9.0	4.7	7.8	7.7	8.9	6.1	4.9	3.86
Number of kernels	456	529	295	58	49	468	462	396	167	596	460	285	301	45	13.10
Thousand-kernel weight (g)	41.8	31.4	45.2	29.8	31.7	38.7	29.4	40.9	23.0	38.1	30.3	38.5	26.8	22.6	2.00

**Table 3:** Nonparametric factorial ANOVA (aligned rank transform, ART) for each parameter. Cells report F-statistics with significance for year, object, and year × object interaction. Significance levels reflect Benjamini–Hochberg FDR-adjusted *p*-values: *** FDR < 0.001; ** FDR < 0.01; * FDR < 0.05.

Marker	Year	Object	Year × Object
*F*_0_	184.95 ***	6.99 ***	4.43 ***
*F*_m_	9.85 ***	5.56 ***	2.27 ***
*F*_v_/*F*_m_	519.45 ***	19.68 ***	11.86 ***
NPQ	23.85 ***	15.45 ***	7.17 ***
qP	7.01**	18.61 ***	4.29 ***
Rfd	1.39	21.42 ***	8.53 ***
Viol	136.92 ***	8.42 ***	3.6 ***
Anth	43.23 ***	3.05 ***	3.15 ***
Lut	31.11 ***	8.19 ***	2.36 ***
Zea	68.19 ***	3.81 ***	4.64 ***
Chl *b*	89.27 ***	9.97 ***	3.43 ***
Chl *a*	30.89 ***	8.1 ***	4.26 ***
β-crt	47.96 ***	4.46 ***	5.42 ***
Chl *a*/*b*	156.39 ***	10.94 ***	3.19 ***
Chl tot.	45.04 ***	8.39 ***	4.1 ***
VAZ	64.14 ***	6.87 ***	3.8 ***
H_2_O_2_	16.69 ***	10.77 ***	6.05 ***
SA	48.22 ***	6.31 ***	5.98 ***
*ScLSD1*	7.12 *	5.38 ***	3.76 ***
*ScAPX1*	57.56 ***	22.62 ***	2.86 **
*ScEDS1*	5.34 *	7.27 ***	3.27 ***
Mass	15.05 ***	68.16 ***	2.79 ***
No. spikes	14.75 ***	16.78 ***	2.19 ***
No. kernels	20.53 ***	59.68 ***	1.52
TKW	43.85 ***	58.3 ***	11.19 ***

## Data Availability

The data presented in this study are available on request from the corresponding author.

## Supplementary Materials

The following supporting information can be downloaded at https://www.mdpi.com/article/10.3390/plants14243746/s1. Figure S1: Variation in example yield and physiological parameters across objects. Figure S2: Correlations between selected traits across years. Figure S3: Principal component biplot of physiological markers measured across rye individuals (N = 201). Figure S4: Average maximum quantum yield of PSII (*F*_v_/*F*_m_) across objects. Figure S5: Breeding scheme illustrating the crosses among the analysed plant lines. Figure S6: Melt-curve specificity for the primers used in qPCR study. Table S1: List of primers used in this study. Table S2: Variance components estimated using linear mixed-effects models (REML). File S1: Table_1_additional_information. File S2: Figure_1_additional_information.
