# Supplementary material for: From Light Harvesting to Grain Filling: Chlorophyll Fluorescence, Pigment Composition, and Oxidative Status as Discrete Yield Determinants in Rye
Source: Plants (Basel). 2025 Dec 9;14(24):3746. doi: 10.3390/plants14243746 (PMC12737187; doi:10.3390/plants14243746)
Supplement: Supplementary file 1 [file plants-14-03746-s001.zip › plants-3982885-supplementary.pdf]

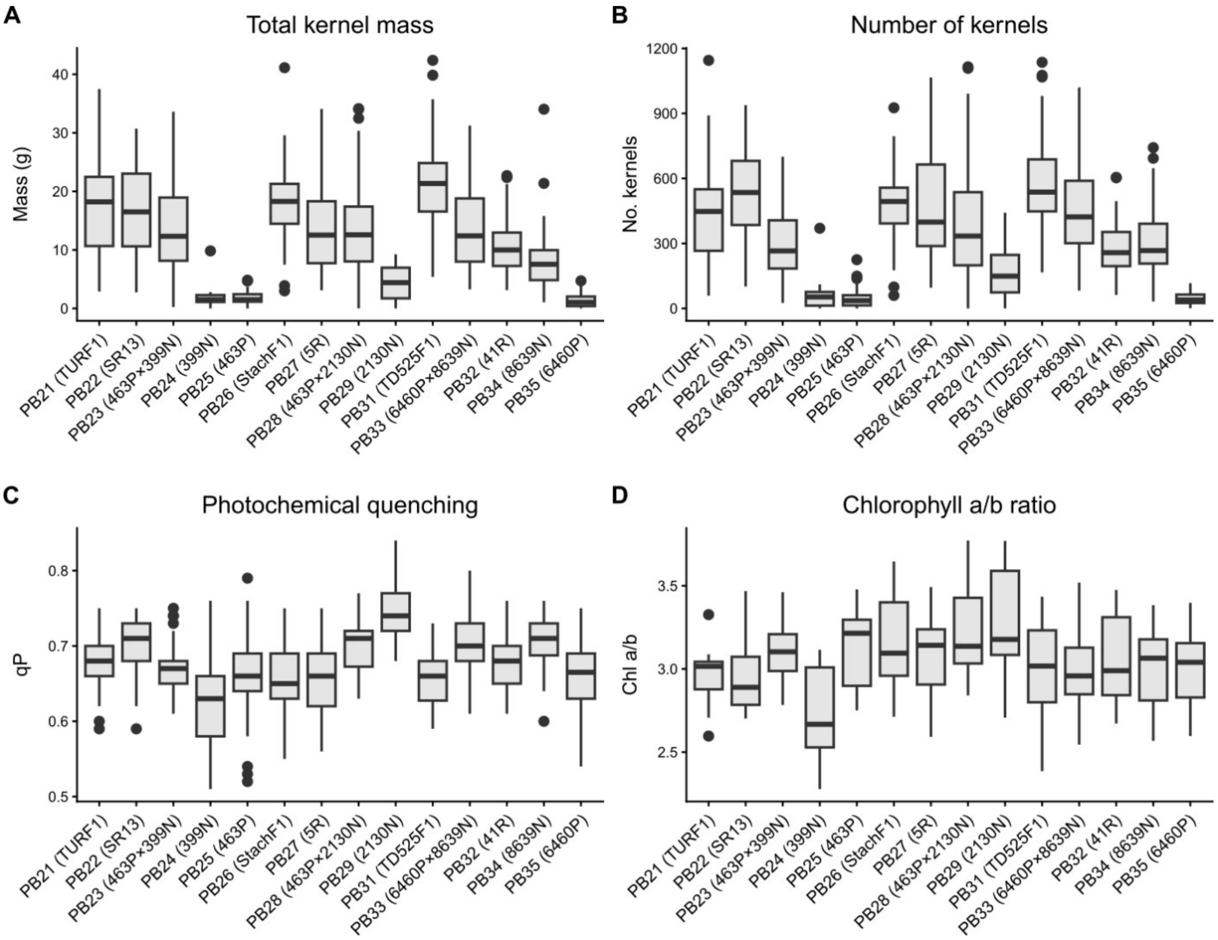

Figure S1. Variation in example yield (A–B) and physiological (C–D) parameters across objects. Boxplots show per-object distributions: the central line marks the median, boxes denote the interquartile range (IQR), whiskers extend to 1.5× IQR from the quartiles, and points beyond the whiskers indicate observations outside this range.

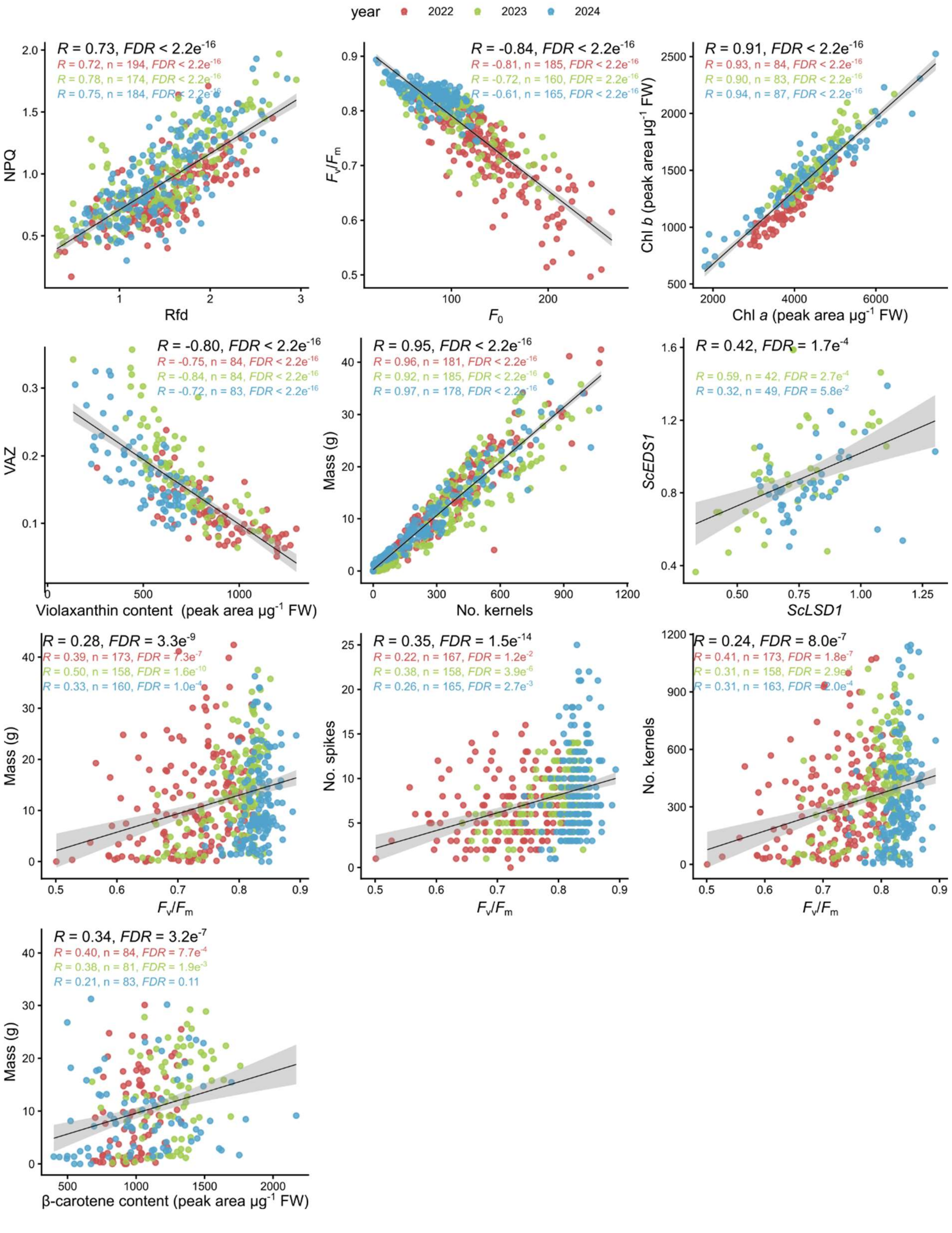

Figure S2. Correlations between selected traits across years. Points represent individual observations, color-coded by year. Black lines show linear regression fits with 95% confidence intervals. Spearman's rank correlation coefficients (*R*) and FDR-adjusted significance values (Benjamini–Hochberg) (*FDR*) are reported within each panel (black: combined dataset; coloured: individual years).

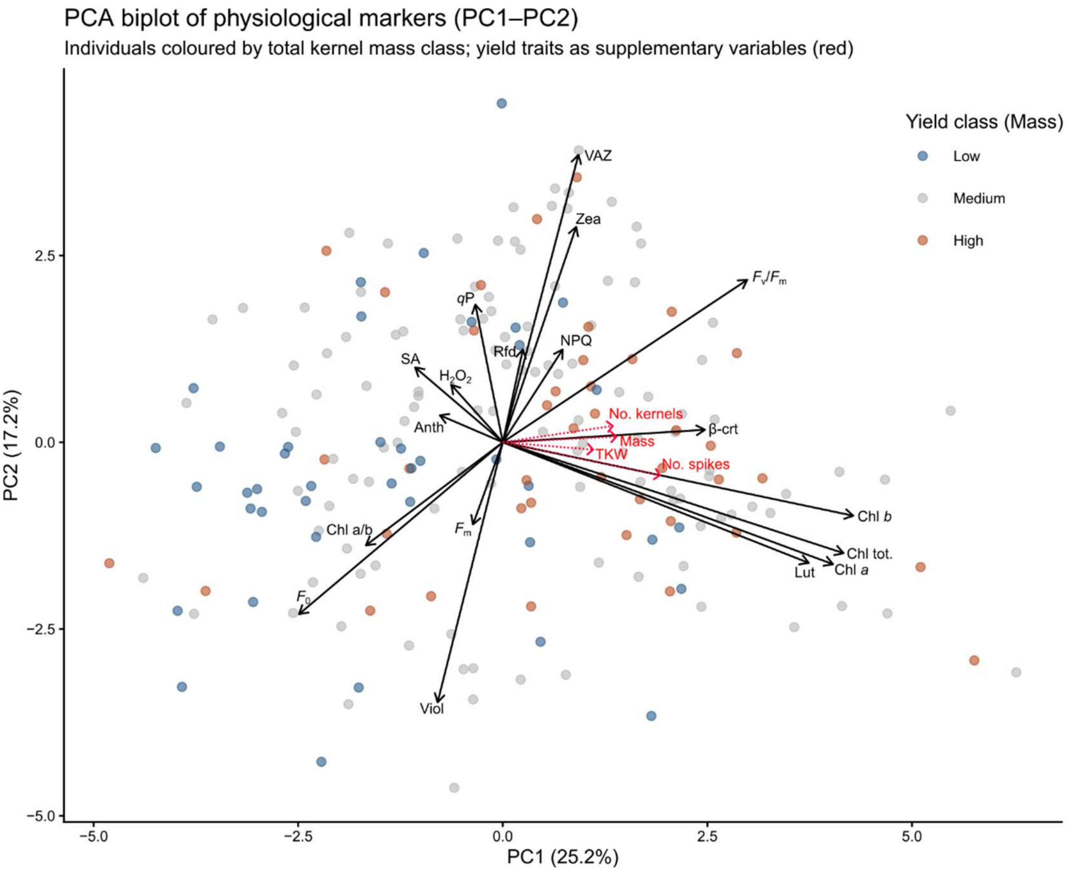

Figure S3. Principal component biplot of physiological markers measured across rye individuals (N = 201). The biplot is showing the distribution of individual plants along the first two principal components (PC1 and PC2), with points coloured according to their total kernel mass class. Vectors represent loadings of physiological (black, solid) and yield (red, dashed) variables. Arrows indicate the direction and relative magnitude of each variable's contribution to the orientation.

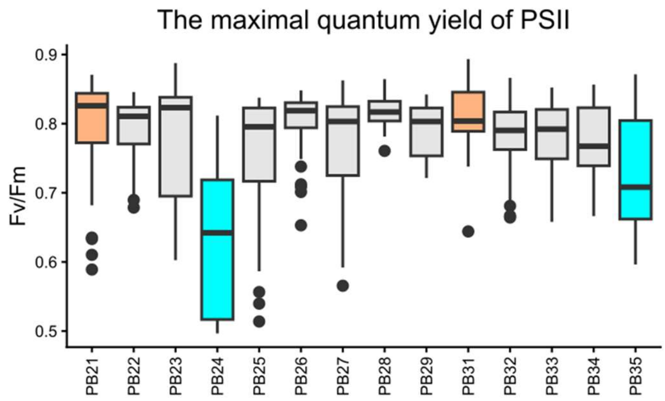

Figure S4. Average maximum quantum yield of PSII (F_v/F_m) across objects. Boxplots represent per-object distributions: the central line indicates the median, boxes show the interquartile range (IQR), whiskers extend to 1.5 × IQR, and points beyond the whiskers denote outliers. Boxplot colours highlight the two objects with the highest total kernel mass (orange) and the two with the lowest total kernel mass (light blue).

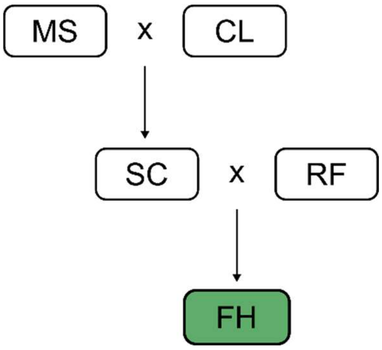

Figure S5. Breeding scheme illustrating the crosses among the analysed plant lines, including final hybrids (FH), restorers of fertility (RF), single cross hybrids (SC), complementary lines (CL), and male sterile lines (MS).

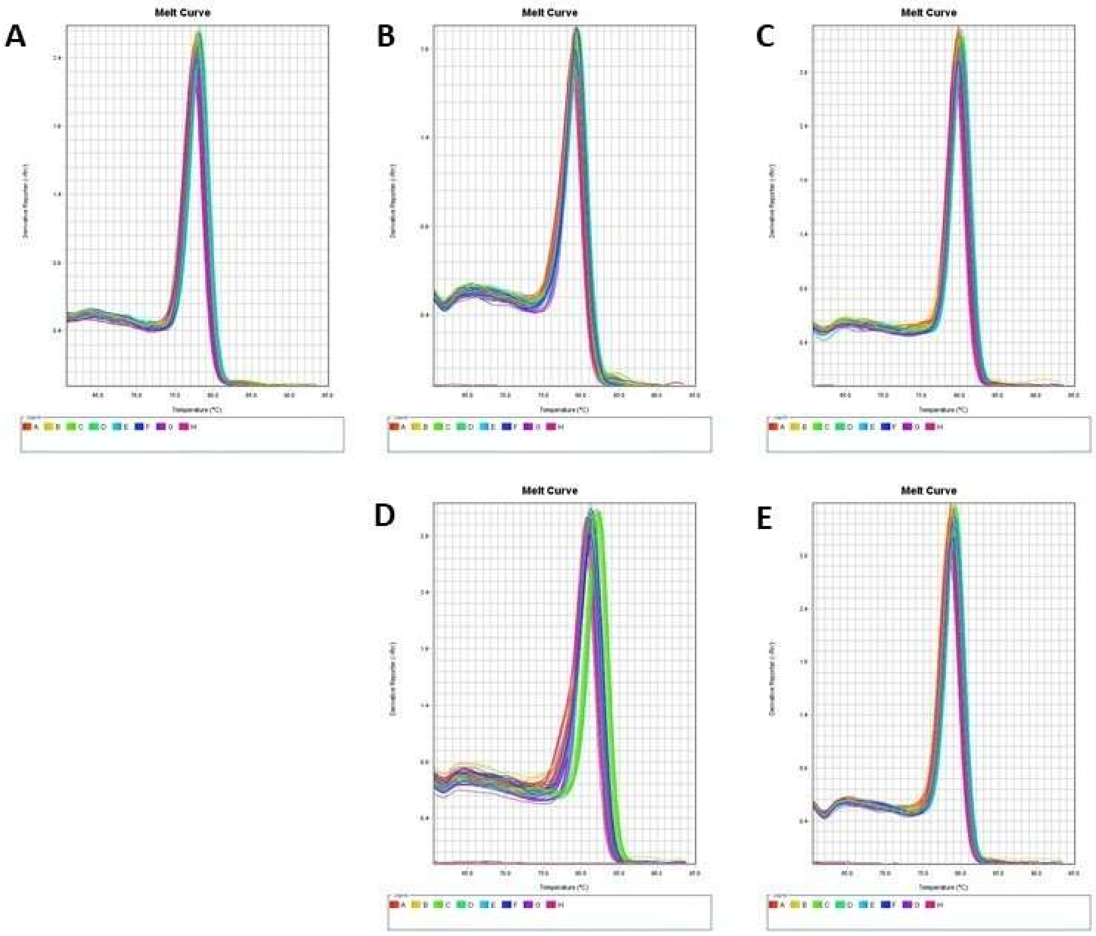

Figure S6. Melt-curve specificity for the primers used in qPCR study. Each graph represents a specific primer: ScACT (A), ScADP-RFa (B), ScLSD1 (C), ScAPX1 (D), ScEDS1 (E).

Table S1. Variance components estimated using linear mixed-effects models (REML). Variance components for year, genotype, genotype × year, and residual were estimated for each trait using a random-effects model. Values shown are the proportion of total variance (%) explained by each component.

Trait	Year	Genotype	Genotype × Year	Residual
Rfd	0.0	17.8	28.6	53.6
Mass	0.7	51.0	3.5	44.7
No. kernels	2.4	49.2	1.3	47.2
No. spikes	5.3	21.4	7.1	66.2
TKW	10.3	20.2	42.4	27.1

Table S2. List of primers used in this study, along with amplicon length and PCR efficiency ± SD for each pair of primers. PCR efficiency was calculated based on linear regression method.

Gene name	Primer F	Primer R	Amplicon length	PCR efficiency ±SD
ScACT	CCCCTTTGAACCCAAAAGCC	GAAAGCACGGCCTGAATAGC	100bp	1,788±0,018
ScADP-RFa	TCTCATGGTTGGTCTCGATG	GGATGGTGGTGACGATCTCT	80bp	1,800±0,023
ScLSD1	ATGCATGCACCAAACGGAAT	ACGTTGCTCACCAGTTTTCC	131bp	1,863±0,021
ScAPX1	CTGAGTGGGGAGAAGGAAGG	CCGCAGCATATTTGTCCACA	97bp	1,869±0,024
ScEDS	CATCATGCCACTGGACATCA	ACAAGCGAATTCCCAACAGG	132bp	1,877±0,020
